# Efficacy of preimplantation genetic testing for aneuploidy in improving pregnancy outcome among couples with azoospermia: study protocol for a multicenter, randomized controlled trial

**DOI:** 10.3389/fendo.2026.1903245

**Published:** 2026-10-07

**Authors:** Yaqiong He, Jiaan Huang, Junhao Yan, Jichun Tan, Zhe Wei, Yun Sun, Yao Lu

**Affiliations:** 1 Department of Reproductive Medicine, Shanghai Key Laboratory for Assisted Reproduction and Reproductive Genetics, Ren Ji Hospital, Shanghai Jiao Tong University School of Medicine, Shanghai, China; 2 State Key Laboratory of Reproductive Medicine and Offspring Health, Centre for Reproductive Medicine, Institute of Women, Children and Reproductive Health, Shandong University, Jinan, China; 3 National Research Centre for Assisted Reproductive Technology and Reproductive Genetics, Shandong University, Jinan, China; 4 Key Laboratory of Reproductive Endocrinology, Ministry of Education, Shandong University, Jinan, China; 5 Research Unit of Gametogenesis and Health of ART-Offspring, Chinese Academy of Medical Sciences (No 2021RU001), Jinan, China; 6 Centre for Reproductive Medicine, Department of Obstetrics and Gynecology, Shengjing Hospital of China Medical University, Shenyang, China; 7 Shanghai Immune Therapy Institute, Shanghai Jiao Tong University School of Medicine-Affiliated Renji Hospital, Shanghai, China

**Keywords:** azoospermia, intracytoplasmic sperm injection, live birth rate, preimplantation genetic testing for aneuploidy, randomized controlled trial

## Abstract

**Background:**

Azoospermia affects up to one in ten infertile couples and represents one of the most challenging conditions in male infertility. Although the development of intracytoplasmic sperm injection (ICSI) enables azoospermic men to achieve biological parenthood, overall success rates remain modest. Preimplantation genetic testing for aneuploidy (PGT-A) is increasingly promoted and used worldwide to improve pregnancy outcomes and enhance the efficiency of ICSI; however, its efficacy for couples with azoospermia remains uncertain. Robust evidence from adequately powered randomized controlled trials (RCTs) is still lacking to determine whether PGT-A can increase the live birth rate as the primary outcome in this context.

**Methods:**

This trial is designed as a prospective, multicenter, open-label RCT. Couples with azoospermia (female age 20–37 years; male age 20–55 years) who intend to undergo ICSI with partner’s sperm will be screened for eligibility. Women who have at least two blastocysts available on day 5 of embryo culture will be enrolled and randomly assigned in a 1:1 ratio to receive either PGT-A or conventional ICSI. A freeze-all strategy with single frozen-thawed blastocyst transfer will be implemented in both groups. The primary outcome is live birth rate.

**Discussion:**

The findings of this trial are expected to provide high-quality evidence regarding the efficacy of PGT-A for couples with azoospermia, helping optimize individualized patient management and improve overall treatment efficiency.

**Clinical trial registration:**

http://www.chictr.org.cn/, identifier ChiCTR2100043421.

## 1 Introduction

Azoospermia, which affects up to one in ten infertile couples, is one of the most challenging conditions in male infertility and assisted reproduction (1–4). Although substantial developments have been made in reproductive technologies in recent decades, and the development of intracytoplasmic sperm injection (ICSI) allows azoospermic men to father biological children, success rates remain modest. According to the International Committee for Monitoring Assisted Reproductive Technologies (ICMART) world report, ICSI yields a pregnancy rate of only about 25-30% and a live birth rate of approximately 20% (5, 6). Poor pregnancy outcomes have translated to high risk of treatment failure recurrently, which causes great distress and frustration to both patients and physicians. Consequently, there is a pressing and unmet need for strategies to improve treatment efficiency and pregnancy outcomes for couples affected by azoospermia.

The underlying reasons for the low efficiency of ICSI are multifactorial. One key factor is that embryo selection for transfer relies primarily on morphology, whereas morphologically normal embryos can be genetically abnormal (7, 8). It has been well known that aneuploidy is the most commonly seen genetic abnormality, which leads to up to 50% of treatment failure, as well as miscarriage (9, 10). To tackle this problem, the technology of preimplantation genetic testing for aneuploidy (PGT-A), working as a test performed on embryos to analyze for chromosomal abnormalities, is increasingly promoted and used worldwide aiming to improve pregnancy outcomes and efficiency of ICSI (11–13). Especially in the past decade, new techniques including array-based comparative genomic hybridization (aCGH) and next generation sequencing (NGS), have invigorated the field (13, 14). These techniques, allowing for comprehensive 24-chromosome screening, have overcome the technical drawback of fluorescence *in situ* hybridization (FISH), and thereby are believed to be beneficial for couples with high risk of aneuploidy (15).

However, clinical evidence regarding the efficacy of PGT-A for couples with azoospermia remains scarce (16–18). Three studies have reported an association between severe male factor (SMF) infertility—including oligozoospermia, asthenozoospermia, teratozoospermia, and azoospermia—and a higher incidence of embryo aneuploidy, with azoospermia specifically linked to an increased risk of mosaicism (19–21). It is important to note that these studies, while compelling, primarily drew their conclusions based on FISH or the analysis of a limited number of chromosomes (2). In contrast, recent results from a large-scale observational cohort study suggested that azoospermia and other severe male factors may impair embryonic competence without affecting the euploidy rate (22). Two small retrospective studies have reported on the use of PGT-A and subsequent pregnancy outcomes in couples with SMF, indicating that PGT-A was associated with a lower rate of early miscarriage or a trend toward increased rates of ongoing pregnancy and live birth per transfer (18, 23). Yet, these studies were limited due to their retrospective design and small sample sizes. High-quality, adequately powered randomized controlled trials (RCTs) focusing on couples with azoospermia are lacking.

## 2 Methods

### 2.1 Study objective

This study aims to evaluate whether PGT-A improves the live birth rate compared with conventional ICSI for couples with azoospermia.

### 2.2 Design and setting

This is a prospective, multi-center, open-label, randomized controlled trial (RCT) conducted at 9 academic hospitals in China. The recruitment period will be from June 2021 to December 2026. All eligible patients will be randomized in a 1:1 ratio to either the PGT-A intervention arm or the conventional ICSI control arm. The study design flowchart is shown in **Figure 1**.

**Figure 1:**
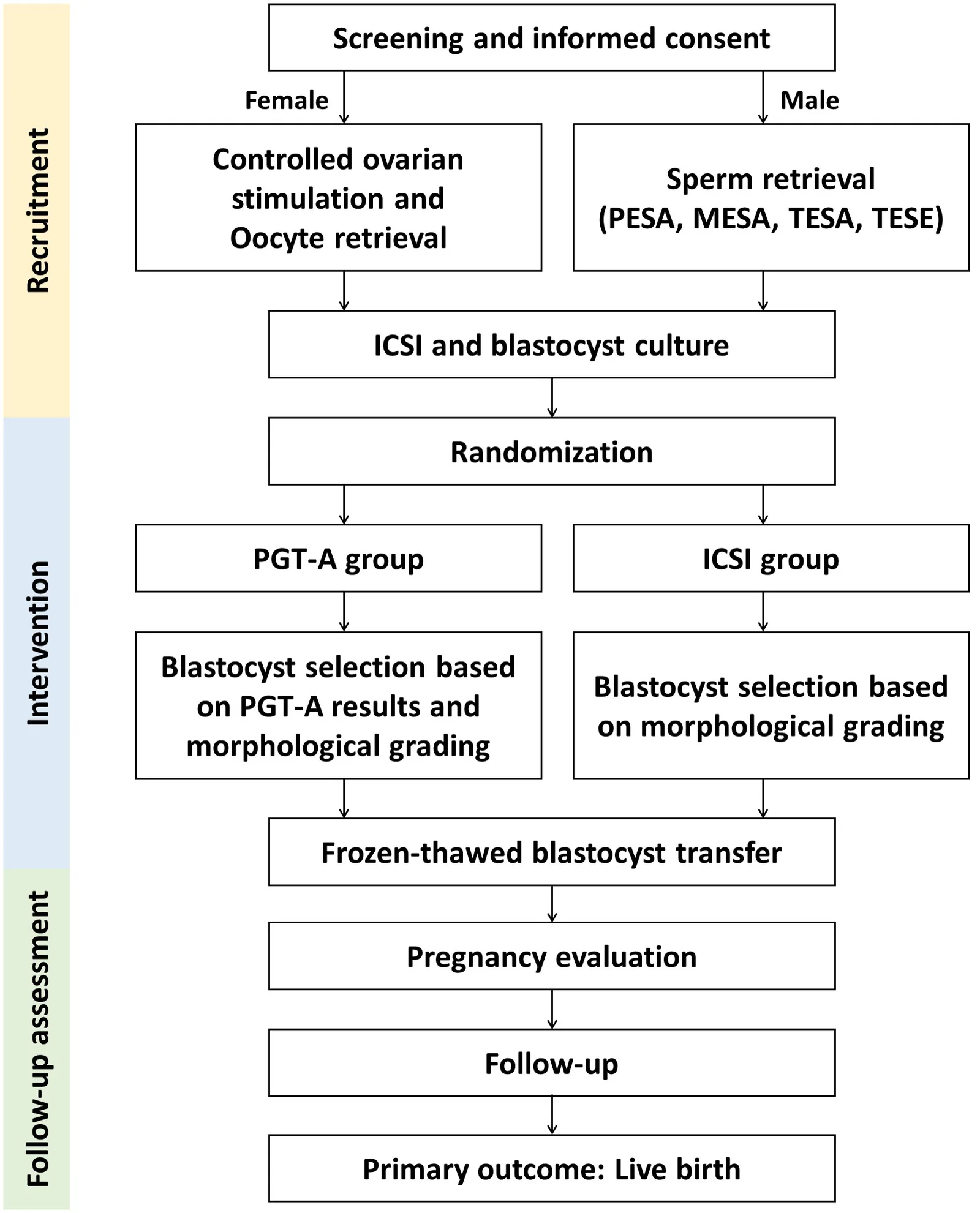
ICSI, intracytoplasmic sperm injection; MESA, microsurgical epididymal sperm aspiration; PESA, percutaneous epididymal sperm aspiration; PGT-A, preimplantation genetic testing for aneuploidy; TESA, testicular sperm aspiration; TESE, testicular sperm extraction. Eligible participants will be randomized into one of the two parallel groups in a 1:1 ratio: the PGT-A group or the ICSI group. In the PGT-A group, all eligible blastocysts will undergo trophectoderm biopsy followed by PGT-A. Blastocysts selected for transfer will be determined based on both PGT-A results and morphological grading. In the ICSI group, all eligible blastocysts will be vitrified per local routine. Blastocysts selected for transfer will be determined solely according to morphological grading.

The study protocol was approved by the ethics committee of Ren Ji Hospital, Shanghai Jiao Tong University School of Medicine (reference number KY2021-002). The trial was registered in the Chinese Clinical Trial Registry (http://www.chictr.org.cn/. Registration number ChiCTR2100043421). Ren Ji Hospital, Shanghai Jiao Tong University School of Medicine, serves as the sponsor of this investigator-initiated trial and bears overall responsibility for the initiation, management, and quality oversight of the trial, including trial design, regulatory and ethical compliance, safety oversight, data governance, and monitoring. The Data Coordinating Center (DCC) is a functional unit established by the sponsor to coordinate data management, monitoring, and safety reporting across participating centers in an independent and standardized manner. The trial will be conducted and reported in accordance with the Consolidated Standards of Reporting Trials (CONSORT) guidelines. Prior to enrollment, a research investigator will fully explain the study procedures to each participant. Written informed consent must be obtained from all participants before they take part in the study.

### 2.3 Eligibility criteria

#### 2.3.1 Inclusion criteria

1. Male to be aged 20–55 years old and female to be aged 20–37 years old;
2. Couples with azoospermia;
3. Couples undergoing intracytoplasmic sperm injection with partner’s sperm;
4. Couples with 2 or more blastocysts on Day 5;
5. Be capable of giving informed consent.

#### 2.3.2 Exclusion criteria

1. Women or their partner with abnormal chromosome karyotype (not including chromosome polymorphisms);
2. Couples with history of PGT-A;
3. Women with history of recurrent pregnancy loss, defined by two or more failed clinical pregnancies;
4. Women with history of recurrent implantation failure, defined by three or more failed embryo transfer cycles;
5. Women who have been diagnosed with a pathology affecting endometrial cavity, such as uterine malformation, submucous fibroids, et al.;
6. Women with medical conditions that represent contraindications to assisted reproductive technology and/or pregnancy.

### 2.4 Sample size

According to published data, the live birth rate among azoospermic couples using surgically retrieved sperm range from approximately 18% to 42% (22, 24–26). Based on multicenter retrospective data from our participating study centers, the live birth rate per transfer was approximately 35% among azoospermic couples who underwent conventional ICSI followed by frozen-thawed single blastocyst transfer. For the present study, we hypothesize that adding PGT-A to select a single euploid embryo will increase the live birth rate from 35% (with conventional ICSI) to 50% (with PGT-A). A total of 534 subjects is estimated to provide 90% power with a two-sided type-1 error of 0.05 to detect an absolute difference of 15% between the PGT-A group and the ICSI group in the rate of live birth, allowing for a dropout rate of 15%. Reasons for dropout include failure to complete embryo transfer within 1 year post-randomization, loss to follow-up, withdrawal of consent, spontaneous conception, and other unforeseen circumstances.

### 2.5 Randomization and allocation concealment

Eligible subjects will be randomly assigned to one of two study arms using a computer-generated dynamic block randomization sequence (block sizes of 2, 4, and 6). The sequence was generated by biostatisticians at the DCC using SAS software version 9.2 (SAS Institute, Cary, NC) and was uploaded to a secure online randomization system by DCC staff prior to study initiation. The DCC staff will keep the sequence strictly confidential, ensuring that the study investigators remain unaware of upcoming group allocations. Randomization will be stratified by azoospermia type (obstructive or non-obstructive) and by study site. On day 5 of embryo culture, authorized investigators will log into a password-protected account to retrieve the allocation information for eligible subjects. This will be an open-label trial, as masking embryo biopsy is neither feasible nor ethical.

### 2.6 Recruitment and screening

Potential participants will be recruited from the andrology and reproductive clinics at the participating centers. A screening visit will be scheduled, during which comprehensive information regarding the trial protocol and study procedures will be provided. Eligible couples who express interest will provide written informed consent and then undergo a comprehensive evaluation. This will include a review of their medical history, which will specifically document detailed reproductive history, past infections, hormonal profiles, surgical history (e.g., hernia repair, scrotal or pelvic surgeries), and exposure to toxins, radiation, or chemotherapy.

**Table 1** (SPIRIT diagram) is a schedule of the study process including enrollment, interventions, and assessment. This protocol refers to the SPIRIT Reporting Guidelines (27).

**Table 1:** SPIRIT diagram for schedule of enrollment, interventions, and assessments.

	Enrolment	Pre-allocation	Allocation	Post-allocation							Close-out
Content	Screening and baseline assessment	Oocyte and sperm retrieval	Blastocysts vitrification	Embryo transfer (ET)	Biochemical pregnancy evaluation	Clinical pregnancy evaluation	12-week gestation follow-up	28-week gestation follow-up	37-week gestation follow-up	Delivery follow-up	Postpartum follow-up
Timepoint	T0	T1	T2	T3	T4	T5	T6	T7	T8	T9	T10
	-3 months	-5 days	0 day	2–12 months	12–15 days after ET	5 weeks after ET	12 weeks of gestation	28 weeks of gestation	37 weeks of gestation	9 months after ET	6 weeks after delivery
Enrolment											
Eligibility screen	×	×	×								
Informed consent	×										
Allocation			×								
Interventions											
PGT-A			×								
ICSI			×								
Assessments											
Baseline data	×	×									
Physical examination	×										
Laboratory tests	×	×		×	×						
Ultrasonography	×			×		×	×	×	×		
Embryo quality			×	×							
Pregnancy test					×						
Pregnancy outcomes						×	×	×	×	×	
Fetus information							×	×	×	×	
Neonatal information										×	×
Adverse events		×	×	×	×	×	×	×	×	×	×

### 2.7 ICSI procedures

#### 2.7.1 Controlled ovarian stimulation and oocyte retrieval

All participants will receive standard COS treatment at each study site. COS protocol will be individualized based on the patient’s age, ovarian reserve, weight, and the physician’s recommendation. Final oocyte maturation will be triggered with human chorionic gonadotropin (hCG) and/or GnRHa when at least one follicle(s) reaches 18 mm in diameter. Transvaginal ultrasound-guided oocyte retrieval will then be performed 34 to 37 hours thereafter.

All participants will be monitored for ovarian hyperstimulation syndrome (OHSS) during ovarian stimulation and after oocyte retrieval. OHSS will be graded as mild, moderate, or severe according to the Golan criteria. Mild and moderate OHSS will be managed with outpatient follow-up, symptomatic treatment, and intravenous fluid support as needed, while severe OHSS will be managed in hospital and reported as serious adverse events within 24 hours.

#### 2.7.2 Azoospermia type and sperm retrieval

The azoospermia type (obstructive or non-obstructive) will be confirmed by experienced andrologists at each study site following a comprehensive diagnostic workup (3), which may include but is not limited to detailed medical history taking, reproductive hormone profiling, scrotal ultrasonography, chromosomal karyotyping, and additional genetic testing when clinically indicated. Sperm will be retrieved via percutaneous epididymal sperm aspiration (PESA), microsurgical epididymal sperm aspiration (MESA), testicular sperm aspiration (TESA), or testicular sperm extraction (TESE) from the epididymis or testicles. The timing and retrieval methodwill be individualized based on clinical indications, physician’s recommendation and patient preference. All relevant information—including the type of azoospermia, surgical method for sperm retrieval, sperm origin, and use of fresh versus frozen-thawed sperm—will be recorded in the case report forms (CRFs).

#### 2.7.3 ICSI and embryo culture

Standard ICSI will be performed 4–6 hours after oocyte retrieval using either fresh or frozen-thawed spermatozoa. Fertilization will be evaluated 16–18 hours post-insemination. All embryos will be cultured *in vitro* to the blastocyst stage. Cleavage embryos will be scored according to Puissant criteria on day 3 (28). Blastocysts will be graded according to Gardner’s criteria on day 5 and day 6 (29).

### 2.8 Intervention

Participants who have two or more blastocysts graded 4BC or better on day 5 will be randomized into one of the two groups:

- PGT-A group: Trophectoderm (TE) biopsy will be performed by experienced embryologists using a laser-assisted approach on fully expanded day 5 or day 6 blastocysts. Approximately five TE cells, located away from the inner cell mass (ICM), will be gently biopsied. Post-biopsy embryo survival will be recorded as a success indicator. The patient’s name, medical record number, and embryo identification number will be clearly annotated to ensure full traceability of all specimens. All biopsied blastocysts will then be vitrified. NGS will be used for PGT-A testing in accordance with standard protocols.
  The reporting resolution will be 30–70% for mosaicism and ≥ 4 Mb for subchromosomal gains and losses. Results will be reported as “euploid” (no chromosomal abnormality detected), “aneuploid” (whole-chromosome or segmental aneuploidy), “mosaic” (partial-chromosome aneuploidy), or “no result” (amplification failure or uninformative outcome). The order of embryo transfer will be determined by both NGS results and morphological grading. Specifically: When multiple euploid embryos are available, transfer order will be determined by morphological grading. When no euploid embryos are available for a participant, transfer of mosaic embryos will be permitted. Selection of mosaic embryos will follow recommendations from clinical guidelines and committee statements on mosaic embryo transfer. No-result embryos will be treated as untested and transferred according to morphological grading.
- ICSI group: All eligible blastocysts will be vitrified according to local routine. The order of embryo transfer will be determined by morphological grading alone.

#### 2.8.1 Quality control for embryo biopsy and PGT-A

All participating centers are academic hospitals officially approved to perform TE biopsy and PGT-A, hold a valid PGT practice license issued by the provincial health authority, and use only NMPA-approved (Class III) PGT-A reagents and sequencing platforms. Each PGT-A laboratory holds a valid clinical gene amplification laboratory certificate and has a qualified team, including clinical geneticists, certified laboratory technicians, and personnel with expertise in bioinformatics and result interpretation.

To improve consistency across all participating centers, all embryologists will complete standardized protocol training prior to study initiation and only those with more than 3 years of experience are permitted to perform biopsies, thereby minimizing iatrogenic embryo damage. Each center follows a standardized in-house quality control protocol for biopsy and sample handling. Although PGT-A is performed locally at each center, all centers have completed standardized pre-clinical validation, including mock biopsies and NGS platform testing using reference DNA standards with known aneuploidy profiles, and all devices must meet predefined performance specifications. The performance of embryo biopsy and PGT-A will be monitored through review of biopsy success rates and inconclusive rates throughout the study period.

### 2.9 Frozen-thawed embryo transfer

Endometrial preparation will be performed using either an artificial or an ovulatory cycle, based on the participant’s characteristics and the physician’s recommendation, beginning after the second menses following oocyte retrieval. This interval will facilitate full recovery from COS and allow adequate time to receive PGT-A test results. Once the endometrial thickness is adequate, luteal phase support (LPS) will be added according to local routine. A single frozen-thawed blastocyst will be transferred in both groups. The embryo transfer procedure will be carried out under transabdominal ultrasound guidance with a full bladder. All medications used for endometrial preparation and LPS will be documented.

Serum hCG levels will be measured 12–17 days after embryo transfer. If pregnancy is confirmed, LPS will be continued until 10–12 weeks of gestation per clinical routine. All pregnancies will be followed through the end of pregnancy. If pregnancy is not achieved or if pregnancy loss occurs after the first blastocyst transfer, subsequent embryo transfer will be recommended. All embryos transferred within one year after randomization will be followed.

### 2.10 Follow-up protocol

The first follow-up will occur at 12 weeks of gestation. Any first-trimester pregnancy complications (including but not limited to spontaneous abortion, ectopic pregnancy, vaginal bleeding, or hyperemesis gravidarum) will be documented through a review of medical records or via telephone contact.

The second pregnancy follow-up will be at 28 weeks of gestation. Information on any second-trimester complications (including but not limited to abortion, prenatal screening results, gestational diabetes, or incompetent cervix) will be collected through a telephone interview.

The third pregnancy follow-up will be at 37 weeks of gestation. Information on any third-trimester pregnancy complications (including but not limited to preterm labor, intrauterine growth restriction, preeclampsia/eclampsia, premature rupture of membranes, placental abruption, or amniotic fluid abnormalities) will be collected through a telephone interview.

The fourth follow-up time point will be at delivery. Participants will be required to notify investigators about the time of delivery. The delivery-related information (including gestational age, mode of delivery, placenta abnormality, and/or delivery complications) and neonatal information (including gender, birth weight, birth defects) will be obtained using designated forms or by reviewing obstetric and neonatal medical records.

Fifth and final follow-up will occur at 6 weeks postpartum. Postpartum information regarding maternal complications (including but not limited to infection, late postpartum hemorrhage, and postpartum depression) and infant complications (including but not limited to neonatal jaundice, neonatal infection, neonatal respiratory distress syndrome, neonatal death, and neonatal hospitalization) will be collected via telephone follow-up or by reviewing medical records.

Participants will receive standard maternity care in accordance with routine practice, alongside the study-specific follow-up. Adverse events and concomitant medications will be collected and recorded at each visit throughout the trial. Any participants who withdraw or are lost to follow-up will be recorded as such.

### 2.11 Discontinuation criteria

1. The participant becomes pregnant spontaneously;
2. The participant requests to withdraw from the clinical trial. The participant is free to withdraw from the study at any time without it affecting their future treatment.

### 2.12 Outcome and outcome assessments

#### 2.12.1 Primary outcome

The primary outcome will be live birth after the first blastocyst transfer, defined as the delivery of any newborn(s) with signs of life at or beyond 28 weeks of gestation.

#### 2.12.2 Secondary outcomes

The secondary outcomes will include biochemical pregnancy, clinical pregnancy, implantation, pregnancy loss, and cumulative live birth. Biochemical pregnancy is defined as a serum β-hCG level ≥10 IU/L measured 12–15 days after embryo transfer. Clinical pregnancy is defined as the detection of an intrauterine gestational sac via ultrasound 28–35 days after embryo transfer. Implantation rate is calculated as the total number of intrauterine gestational sacs detected by transvaginal ultrasound divided by total number of embryos transferred. Pregnancy loss is defined as a pregnancy that results in a spontaneous or therapeutic abortion at any point during gestation. Cumulative live birth rate is calculated as the number of women who achieve a live birth resulting from any embryo transfer within one year after randomization, divided by the total number of women randomized to each group (30). Safety outcomes will include maternal and neonatal complications, birth weight, and congenital anomalies. Additionally, cost-effectiveness outcomes including treatment costs, time to live birth, and number of transfer cycles to live birth will be assessed as exploratory endpoints.

### 2.13 Adverse events

An adverse event (AE) is defined as any untoward medical occurrence that arises during the trial period, irrespective of whether it is considered related to the study intervention. A serious adverse event (SAE) is defined as any event occurring during the subject’s participation in this trial that meets one or more of the following criteria: death; a life-threatening condition; persistent or significant disability or incapacity; requirement for inpatient hospitalization or prolongation of an existing hospitalization; neonatal death up to 42 days post-delivery; a congenital abnormality or birth defect; or any other condition deemed serious by the principal investigator (PI) based on appropriate medical judgment.

All AEs and SAEs will be thoroughly evaluated and documented by the site investigator. The following reporting timelines will be followed: 1) From the site investigator to the PI and DCC: The site investigator will report any SAE to the PI and the DCC within 24 hours of first becoming aware of the event. 2) From the PI/sponsor to the Ethics Committee: Upon receiving a report, the PI will assess the seriousness of the event and its potential causal relationship to the study intervention. All SAEs will be reported to the Ethics Committee (EC) of Ren Ji Hospital, which serves as both the sponsor and the reviewing EC for this investigator-initiated trial. For fatal or life-threatening events, the PI will notify the EC within 24 hours of first becoming aware of the event, followed by a complete written report within 5 working days. For non-fatal, non-life-threatening SAEs, the PI will submit a written report to the EC within 10 working days of first becoming aware of the event. 3) Reporting to the DSMB: All SAEs will be reported to the DSMB through the DCC. For fatal or life-threatening SAEs, the DSMB will be notified within 24 hours. Affected participants will receive appropriate medical care and follow-up in accordance with standard clinical practice. The PI will maintain comprehensive documentation for each SAE, including the initial report and any actions taken.

### 2.14 Protocol deviations

A protocol deviation is defined as any departure from the approved protocol. Deviations will be classified as major or minor according to their impact on participant safety, data integrity, or trial validity. Major deviations (e.g., eligibility violations, crossovers, or failure to receive the assigned intervention) will be reported to the sponsor and DCC within 5 working days of identification and, where they may affect participant safety or data integrity, to the EC and DSMB. Minor deviations will be summarized and reported periodically. All deviations will be documented thoroughly in the CRFs. Major deviations will be excluded from the per-protocol analysis, while the primary analysis will remain based on the intention-to-treat principle.

### 2.15 Data management

Before the trial commences, all investigators, including physicians, nurses, and research assistants, will participate in a training course to ensure a thorough understanding of the study protocol, as well as the accuracy of outcome assessments and data collection. Each investigator will be provided with a copy of the protocol and the standard operating procedures. All research data will be collected using standardized case report forms (CRFs) designed to prevent the tracing of participants’ personal information. The DCC is responsible for data monitoring. DCC staff will routinely verify the completeness, accuracy, and integrity of the data, to ensure the quality of the data collected.

### 2.16 Trial monitoring

An independent Data and Safety Monitoring Board (DSMB), composed of physicians and statisticians, will be established prior to trial commencement. The DSMB will monitor trial progress and participant safety at regular intervals throughout the study. The DCC will prepare DSMB reports, including actual versus projected enrolment, randomization progress, overall dropout and protocol deviation rates, AEs, SAEs, and updated primary outcome measures. After reviewing each report, the DSMB will evaluate the need for further benefit–risk assessment and assess whether the trial should continue as planned, be modified, or terminate early due to safety concerns, futility or overwhelming evidence of benefit. Although this trial does not include a formal interim analysis, the DSMB has the authority to recommend early termination, temporary suspension of enrollment, or protocol modification if it identifies a safety signal that materially alters the benefit–risk balance. All DSMB members are independent of the trial investigators and sponsor and declare no competing interests.

A risk-based monitoring strategy will be implemented by the DCC. Each center will receive an initiation visit, routine monitoring at least once per year during enrollment, and a close-out visit, with frequency adjusted due to enrollment, protocol deviations, or data quality issues. *Ad hoc* visits may be triggered by SAEs, suspected misconduct, or persistent non-compliance. Monitoring will be primarily onsite, supplemented by remote review of CRF data and safety reports. Source data verification (SDV) will be performed in a risk-based and targeted manner. Critical data (including eligibility, randomization, primary and key secondary outcomes, SAEs, and key protocol deviations) will be verified for all randomized participants.

### 2.17 Statistical analysis

Normally distributed continuous variables will be presented as mean ± standard deviation (SD), while non-normally distributed continuous variables will be presented as median and interquartile range (IQR). Between-group differences will be assessed using independent-samples t-tests or Wilcoxon rank-sum tests, as appropriate. Categorical variables will be presented as frequencies and percentages. Between-group differences will be evaluated using Pearson’s chi-square test or Fisher’s exact test, as appropriate. A two-sided alpha level of 0.05 will be considered statistically significant.

The primary analysis will be conducted in accordance with intention-to-treat (ITT) principles. The primary outcome, live birth rate, will be compared between the two groups using Pearson’s chi-square test. For the primary analysis, women lost to follow-up will be considered as not having achieved a live birth. A sensitivity analysis using multiple imputation by chained equations (MICE) under the missing-at-random assumption will be conducted as needed. Secondary outcomes, including rates of biochemical pregnancy, clinical pregnancy, implantation, pregnancy loss, and cumulative live birth, as well as the incidence of maternal and neonatal complications and congenital anomalies, will be analyzed using Pearson’s chi-square test or Fisher’s exact test, as appropriate. Between-group differences in birth weight, time to live birth, embryo transfers to live birth, and treatment costs will be compared using t-tests or Wilcoxon rank-sum tests, as appropriate.

As a secondary analysis, a per-protocol analysis will be performed among participants who complied with the trial protocol. Major protocol noncompliance shall be defined as any of the following circumstances: failure to adhere to the assigned study intervention per trial protocol, identification of eligibility violations after randomization, and premature withdrawal before study completion. Additionally, Kaplan-Meier curves will be generated for cumulative live birth events in the PGT-A and ICSI groups and compared using the log-rank test as a sensitivity analysis. Live birth resulting from any embryo transfer conducted within the 12-month follow-up period will be designated as the primary positive endpoint for CLBR. Participants who exhaust all vitrified embryos without achieving a live birth will be administratively censored at the time of their last embryo transfer. Subgroup analyses will be performed according to age, type of azoospermia (obstructive or non-obstructive), sperm origin (testicular or epididymal) and sperm type (fresh or frozen).

## 3 Discussion

This prospective multicenter randomized controlled trial is designed to compare pregnancy outcomes following NGS-based PGT-A versus conventional morphology-based embryo selection among couples with azoospermia, aiming to provide high-quality evidence for the rational application of PGT-A in this specific patient population. A total of 534 couples will be recruited across nine hospitals in China, and we hypothesize that PGT-A improves live birth rates for azoospermic patients.

Azoospermia is a major cause of male infertility and is categorized into obstructive (OA) and non-obstructive azoospermia (NOA). Although ICSI enables azoospermic men to conceive biological offspring via surgically retrieved gametes, studies have indicated that sperm harvested from the epididymis or testis may carry a higher risk of embryonic aneuploidy than ejaculated sperm (21, 31, 32). Thus, PGT-A has often been proposed and offered for this patient group (13). Nevertheless, many academic societies acknowledge that the current evidence is insufficient to support the routine use of PGT-A in such cases due to limited available data (33, 34). Moreover, since OA and NOA have fundamentally distinct pathogeneses, the magnitude of clinical benefit conferred by PGT-A may differ between these two subtypes (3). In patients with OA, intrinsic testicular spermatogenesis remains intact. Prolonged sperm retention within the epididymis resulting from genital tract obstruction predominantly leads to elevated sperm DNA fragmentation and epigenetic perturbations (35, 36). In contrast, NOA is characterized by primary testicular spermatogenic failure, manifesting as germ cell maturation arrest and impaired meiotic homologous recombination, which may substantially increase the risk of chromosomal non-disjunction during meiosis (32, 37). Accordingly, this trial employs stratified randomization based on azoospermia type and predefines corresponding subgroup analyses. However, subgroup analyses should be designated exploratory and hypothesis-generating only, given the limited sample size within each stratum.

To the best of our knowledge, this is the first multicenter randomized trial to evaluate the efficacy of PGT-A in couples with azoospermia. Female age is restricted to 20–37 years to minimize confounding effects from advanced maternal age. Consequently, the findings of this trial cannot be generalized to women of older reproductive age. Patients with two or more blastocysts will be enrolled, as this group is most likely to benefit from further embryo selection, whereas those with only one blastocyst typically opt for immediate transfer rather than genetic screening (38–40). A universal freeze-all strategy with single blastocyst transfer will be applied to improve outcome comparability between study groups.

The primary outcome is live birth rate, which is recognized as the most patient-centered and clinically meaningful endpoint for infertility treatment trials (41). This outcome also enables comprehensive monitoring and documentation of all pregnancy and neonatal complications throughout the study, further informing the safety profile of PGT-A. The secondary outcomes include cumulative live birth rate per oocyte retrieval cycle, an important metric for assessing overall reproductive success, particularly in the context of PGT-A (30, 42). In addition, this trial evaluates cost-effectiveness parameters, including treatment costs and time to live birth. Overall, the findings will provide robust evidence regarding the efficacy and safety of PGT-A for azoospermic couples, helping optimize patient care and improve treatment efficiency in routine clinical practice.

We acknowledge that the open-label study design may introduce inherent risks of assessment bias. To mitigate this potential bias, multiple strategies have been implemented throughout the trial. First, all primary and secondary endpoints are objective clinical outcomes evaluated according to unified, predefined diagnostic criteria. Second, consistent standard operating procedures (SOPs) are applied across all participating centers to standardize follow-up schedules and outcome recording. Third, all statistical analyses will strictly adhere to the pre-approved Statistical Analysis Plan (SAP). Finally, an independent DSMB was established before trial commencement to regularly supervise data quality, thereby minimizing operational bias and selective outcome reporting bias.
